# Internet-Based Cognitive Behavioral Therapy for Patients Reporting Symptoms of Anxiety and Depression After Myocardial Infarction: U-CARE Heart Randomized Controlled Trial Twelve-Month Follow-up

**DOI:** 10.2196/25465

**Published:** 2021-05-24

**Authors:** Sophia Monica Humphries, John Wallert, Fredrika Norlund, Emma Wallin, Gunilla Burell, Louise von Essen, Claes Held, Erik Martin Gustaf Olsson

**Affiliations:** 1 Clinical Psychology in Healthcare Department of Women's and Children's Health Uppsala University Uppsala Sweden; 2 Centre for Psychiatry Research Department of Clinical Neuroscience Karolinska Institute Stockholm Sweden; 3 Centre for Alzheimer Research Department of Neurobiology, Care Sciences and Society Karolinska Institute Stockholm Sweden; 4 Department of Psychology Uppsala University Uppsala Sweden; 5 Family Medicine and Preventative Medicine Department of Public Health and Caring Sciences Uppsala University Uppsala Sweden; 6 Department of Medical Sciences, Cardiology Uppsala Clinical Research Centre Uppsala University Uppsala Sweden

**Keywords:** myocardial infarction, iCBT, psychological treatment, cardiovascular health, cognitive behavior therapy, internet, cardiovascular, infarction, treatment, anxiety, depression

## Abstract

**Background:**

The U-CARE Heart trial was one of the first randomized controlled trials to evaluate the effect of internet-based cognitive behavioral therapy on self-reported symptoms of anxiety or depression for patients with a recent myocardial infarction. While the effects of internet-based cognitive behavioral therapy on Hospital Anxiety and Depression Scale (HADS) scores at 14 weeks postbaseline were not significant, in this study, we investigated possible long-term effects of treatment.

**Objective:**

The aim of this study was to evaluate the long-term effectiveness of internet-based cognitive behavioral therapy on self-reported symptoms of anxiety and depression in patients 12 months after a myocardial infarction and to explore subsequent occurrences of cardiovascular disease events.

**Methods:**

Shortly after acute myocardial infarction, 239 patients (33% female, mean age 59.6 years) reporting mild-to-moderate symptoms of anxiety or depression were randomized to 14 weeks of therapist-guided internet-based cognitive behavioral therapy (n=117) or treatment as usual (n=122). Data from national registries were used to explore group differences in clinical outcomes such as cardiovascular disease and cardiovascular-related mortality for a follow-up period of up to 5 years: group differences in HADS total score 1 year post–myocardial infarction, the primary outcome, was analyzed using multiple linear regression. Secondary outcomes, such as HADS anxiety and depression subscales and the Cardiac Anxiety Questionnaire total score (CAQ), which measures heart-focused anxiety, were analyzed in the same way. Multiple imputation was used to account for missing data, and a pooled treatment effect was estimated. Adjusted Cox proportional hazards models were used to estimate hazard ratios (HRs) for data pertaining to registry outcomes.

**Results:**

Both groups reported lower HADS total scores 1 year after myocardial infarction than those at baseline. HADS total scores were not significantly different between the treatment and control groups 1 year after myocardial infarction (*β*=–1.14, 95% CI –2.73 to 0.45, *P*=.16). CAQ was the only measure improved significantly by internet-based cognitive behavioral therapy when compared with treatment as usual (*β*=–2.58, 95% CI –4.75 to –0.42, *P*=.02) before adjusting for multiple comparisons. The composite outcome of nonfatal cardiovascular events and cardiovascular-related mortality did not differ between groups but was numerically higher in the internet-based cognitive behavioral therapy group, who were at slightly greater risk (HR 1.8, 95% CI 0.96 to 3.4, *P*=.07). Adjusting for previous myocardial infarction and diabetes attenuated this estimate (HR 1.5, 95% CI 0.8 to 2.8, *P*=.25).

**Conclusions:**

Internet-based cognitive behavioral therapy was not superior in reducing self-reported symptoms of depression or anxiety compared to treatment as usual at the 1-year follow-up after myocardial infarction. A reduction in cardiac-related anxiety was observed but was not significant after adjusting for multiple comparisons. There was no difference in risk of cardiovascular events between the treatment groups. Low treatment adherence, which might have affected treatment engagement and outcomes, should be considered when interpreting these results.

**Trial Registration:**

ClinicalTrials.gov NCT01504191; https://clinicaltrials.gov/ct2/show/NCT01504191

**International Registered Report Identifier (IRRID):**

RR2-10.1186/s13063-015-0689-y

## Introduction

Myocardial infarction continues to be the leading cause of death worldwide [1]. The risk increases with age and is associated with behavioral factors such as smoking and reduced physical activity [2]. For over one-third of patients after myocardial infarction, symptoms of anxiety, depression, or both remain elevated 3 months after hospital discharge [3], and similar levels have even been reported up to 1 year after myocardial infarction [4]. A preexisting history of mental illness, such as depression, is associated with a 31% increased risk of a recurrent myocardial infarction [5] and a 30% higher mortality rate in the 18 months following myocardial infarction than among those without a history of depression [6]. Mortality rates increase with severity of depression, according to one study [7] and, depressive symptoms are reported to be more prevalent among patients with more cardiovascular risk factors (eg, smoking and obesity) [8]. This combination of increased risk for morbidity following a myocardial infarction and lower reported quality of life [9,10] makes therapy that is aimed at reducing symptoms of anxiety and depression likely a beneficial treatment option. In a recent meta-analysis [11], psychological treatment for patients with coronary heart disease was linked to a reduction in self-reported symptoms of anxiety and depression and reduced risk of cardiac-related mortality compared to treatment as usual.

Web-based interventions offer remote access to treatment which are sometimes otherwise inaccessible [12] and have been evaluated against face-to-face therapy in randomized controlled trials with varying yet promising results [12-14]. Some trials have used therapy conducted online, often in the form of internet-based cognitive behavioral therapy, to treat depression in patients with heart failure [15], cardiovascular disease [16], and with high-cardiovascular disease risk [17], but before the U-CARE Heart trial [18], no study had offered internet-based cognitive behavioral therapy specifically to patients after myocardial infarction. A more recent study [19] of internet-based cognitive behavioral therapy for symptoms of anxiety and depression in patients who had experienced an acute coronary event, including myocardial infarction, demonstrated a strong effect of treatment on reduction of these symptoms that was sustained at a 4-week follow-up.

The U-CARE heart trial [18] was, to the authors’ knowledge, the first randomized controlled trial to offer internet-based cognitive behavioral therapy to patients experiencing mild to moderate self-reported symptoms of anxiety or depression after a myocardial infarction. Posttreatment follow-up at 14 weeks found an overall reduction in symptoms of anxiety and depression, according to the Hospital Anxiety and Depression Scale (HADS) [20] compared to those at baseline, yet no differences were found between the intervention and control group. Long-term treatment follow-up of the study population was intended to gather more information about the sustainability of general improvements in anxiety and depression and whether group characteristics (symptoms of anxiety and depression, myocardial infarction and stroke events, and cardiovascular-related mortality) differed at follow-up. The ultimate goal of a psychological intervention post–myocardial infarction would be, not only to reduce symptoms of anxiety and depression long-term but also, to reduce cardiovascular morbidity and mortality, although this was not the main purpose of the U-CARE Heart trial.

The purpose of this follow-up study was thus to investigate whether patients with mild-to-moderate symptoms of anxiety or depression, self-reported within 2 months following myocardial infarction and treated with internet-based cognitive behavioral therapy, had (1) improvements in self-reported mental health at 12 months after the myocardial infarction, and (2) reduced risk for cardiovascular events including cardiovascular-related mortality, for a period of up to 5 years, compared to those receiving treatment as usual.

## Methods

### Study Design and Participants

This follow-up study (1) evaluated the effect of internet-based cognitive behavioral therapy on self-reported symptoms of anxiety and depression 12 months after the myocardial infarction, and (2) explored whether internet-based cognitive behavioral therapy had an effect on nonfatal cardiovascular events or cardiovascular-related mortality up to 5 years after study inclusion. The study design, procedure, intervention, and results of the posttreatment follow-up of the U-CARE Heart trial have been reported [18,21].

In brief, the U-CARE Heart trial was a multicenter study that recruited participants <75 years of age, reporting symptoms of anxiety or depression (scoring >7 on any of the 2 HADS anxiety and depression subscales) within 3 months following their myocardial infarction. Myocardial infarction was defined according to International Statistical Classification of Disease Tenth Revision (ICD-10) code I21 and diagnosed by a cardiologist. Patients scheduled for coronary artery bypass surgery, with low adherence (such as missing appointments with the cardiac nurse or substance use), or expected to live <1 year, as judged on-site by the recruiting nurse, were not eligible. Patients who met eligibility criteria (n=239) were randomized to receive either internet-based cognitive behavioral therapy (n=117) or treatment as usual (n=122). This study received ethical approval by the regional ethics committee in Uppsala (2011/217) and is registered on ClinicalTrials.gov (NCT01504191).

### Procedure

**Figure 1:**
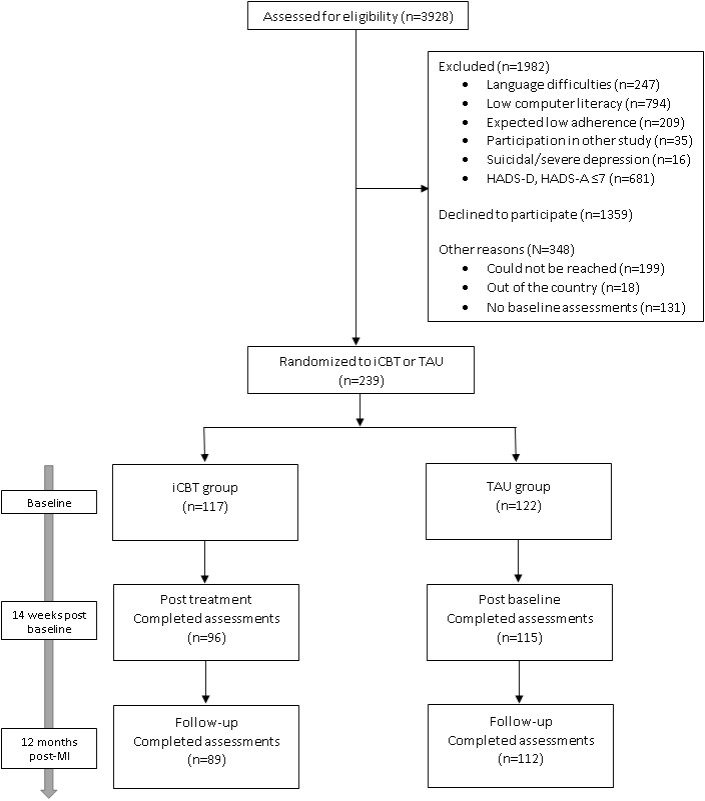
Participation flowchart. iCBT: internet-based cognitive behavioral therapy; TAU: treatment as usual; HADS-D: Hospital Anxiety and Depression Scale–Depression subscale; HADS-A: Hospital Anxiety and Depression Scale–Anxiety subscale; MI: myocardial infarction.

Screening for eligible participants took place across in Sweden from September 2013 to December 2016 in 25 cardiac clinics during routine visits between 1 and 8 weeks following their myocardial infarction. Consenting participants were sent an email and password login to a secure internet-based portal (Multimedia Appendix 1) where they completed baseline assessments and would subsequently receive the internet-based cognitive behavioral therapy (if applicable). Eligibility (Figure 1) was assessed in the baseline questionnaires and those scoring >7 on any or both of the HADS subscales were randomized automatically in the internet-based portal 1:1 to internet-based cognitive behavioral therapy or treatment as usual. The full recruitment procedure has previously been reported [18].

Trial-specific outcomes were measured 14 weeks postbaseline, which corresponded to treatment completion for those in the internet-based cognitive behavioral therapy group, and again at follow-up 12 months after myocardial infarction. Participants in both the intervention and treatment-as-usual groups were sent automatic SMS texts and email reminders asking them to fill in the web-based questionnaires at each observation point and were subsequently reminded via telephone by one of the research staff (blind to treatment allocation) if initially unresponsive. Figure 1 details the patient flow through the study.

### Intervention

The internet-based cognitive behavioral therapy intervention [18,21] delivered via the U-CARE portal (a secure web-portal) was therapist guided and partly customizable. This meant that after a compulsory introduction module, patients could choose their treatment modules. The intervention was developed for cardiac patients and consisted of 11 modules: Introduction, Managing Worry, Fear and Avoidance, Behavioral Activation, Problem Solving, Communication Skills, Applied Relaxation Training, Managing Negative Thoughts, Coping With Insomnia, Values in Life, and Relapse Prevention. Homework assignments were included in each module, and the portal also included a library that was accessible at all times with content and material including media such as videos and informational text.

All participants received standard protocol secondary prevention and cardiac rehabilitation offered by the regional health care system. This usually includes, but is not limited to, preventive medications, education about cardiovascular risk-factors, smoking cessation, organized and tailored physical exercise, and in some areas, access to counseling or psychosocial support.

### Measures

#### Sociodemographic and Psychological Measures

Sociodemographic data were self-reported at baseline. The primary outcome measure was HADS total score, measuring self-reported symptoms of both anxiety and depression (subscales). The HADS is a useful tool in the screening of depression in cardiac patients [22,23], and there is support of its validity for online use [24].The 2 subscales are divided equally between 14 questions, with 7 questions pertaining to each subscale. Questions are answered on a 4-point scale (0-3), with a score >7 on either subscale indicating at least mild symptoms [20].

Secondary outcome measures included the Cardiac Anxiety Questionnaire (CAQ) [25], which assesses self-reported heart-related anxiety; Montgomery-Åsberg Depression Rating Scale–Self-rated (MADRS-S) [26], which measures depression; and the Behavioral Activation for Depression Scale Short–Form (BADS-SF), which assesses depression-related behaviors [27]. In contrast to the other scales, for which a high score denotes more severe symptoms, a high score on the BADS-SF is favorable as it indicates higher activation of change to depression-related behaviors.

#### Register Data

Data on cardiovascular events and mortality during follow-up were obtained from the Patient Registry and the Causes of Death Registry of the National Board of Health and Welfare in Sweden [28]. ICD-10 diagnostic codes were used [29]. A composite cardiovascular event outcome was created comprising hospitalization for any of the following diagnoses between time of randomization and point of data censoring: cardiovascular death (ICD cause of death code I), acute coronary syndrome (ICD codes I20, I21, and I22), heart failure (ICD codes I50 and I11.0), stroke (ICD codes I61, I62, I63, and I64), and revascularization (intervention codes FNG00, FNG02, FNG05, FNG10, and FNC). Background data for medical history were taken from the Swedish Web-System for Enhancement and Development of Evidence-Based Care in Heart Disease Evaluated According to Recommended Therapies (SWEDEHEART) registers [30]: RIKS-HIA (the National Register for Information and Knowledge on Heart Intensive Care Admission) and SEPHIA (the Registry for Secondary Prevention after Heart Intensive Care Admission). These sources of data are linked via a personal identification number that is unique to every registered resident in Sweden, matched to the study cohort with codes, and handled in accordance with current data protection standards.

### Statistical Analysis

Statistical analysis was planned in line with CONSORT (Consolidated Standards of Reporting Trial) before treatment allocation was disclosed and before the trial database was locked.

Main analysis investigated potential differences in HADS total scores 12 months post–myocardial infarction, with subsequent analyses of the HADS anxiety and HADS depression subscale scores. Secondary analyses focused on potential differences between groups in MADRS-S total score, CAQ total score, and BADS-SF total score.

Linear modeling was used to estimate treatment effects. As with the posttreatment analysis [18], analyses were conducted based on intention to treat. Treatment was an independent variable and 12-month follow-up HADS total score as the dependent variable, while controlling for baseline HADS total score, sex, and age. This adjustment for covariates was applied to all analyses. The HADS subscales were analyzed in the same way but only with patients included in the study based on the specific subscale. Thus, it was possible for patients to be included in both analyses for both subscales if they scored >7 on both at baseline.

To account for missing values and analyze according to intention to treat, data were imputed by multiple imputation using chained equations and predictive mean matching [31]. Variables used in the imputation model were the same as those in the posttreatment analysis [18], which were chosen on the basis of potential association with the main outcome and with input from cardiologist and psychologist recommendations. Unless specified, reported results are based on imputed data.

HADS total score sensitivity analyses were conducted on observed data (ie, nonimputed). Supplementary analyses of HADS total score were based on per protocol data, including only those in the model who had completed at least one homework assignment. Only the analyses according to intention to treat (with imputed data) were applied to the secondary outcomes. Effect estimates are reported as pooled adjusted point estimates *β* with 95% confidence intervals. Paired *t* tests were conducted with outcomes pre- vs posttreatment to assess change over time across the full sample. Level of significance, α, for all separate comparisons was set to *P*<.05. For familywise testing of the multiple hypotheses, a Bonferroni-corrected *P* value=.008 (6 hypotheses) was used.

Multivariable Cox regression analysis was performed to estimate adjusted hazard ratios (HR) with 95% confidence intervals with time (in days) to first cardiovascular event as the outcome event. The exposure was treatment group, with time under risk starting from the date of randomization during follow-up and censoring at the time of any cardiovascular event, end of follow-up (December 31, 2018), or the date of noncardiovascular death, whichever came first.

The first participant was randomized in September 2013. The longest period of follow-up spanned 1898 days, and the shortest spanned 719 days; the mean follow-up period was 1139.3 days. Any incidence of cardiovascular death or cardiovascular disease event was used as the primary outcome for time-to-event analysis. There were no missing data for these outcomes. Adjustments for age and sex were performed for higher precision. Posthoc exploratory analyses were conducted with previous myocardial infarction and diabetes as additional covariates since previous medical history of these appeared to be more prevalent in the treatment group. All statistical analyses were performed using R statistical software (version 4.0.2, The R Project).

### Missing Data

Of 239 patients who were enrolled, 24% (28 participants) from the internet-based cognitive behavioral therapy group and 8% (10 participants) from the treatment-as-usual group had missing data on the HADS questionnaire at the 12-month follow-up. In the internet-based cognitive behavioral therapy and treatment-as-usual groups 26% (31 participants) and 9% (11 participants), respectively, had missing data on MADRS-S; 26% (30 participants) and 8% (10 participants), respectively, had missing CAQ values; and 30% (35 participants) and 11% (13 participants) respectively, had missing data on the BADS-SF.

Data were considered missing if (1) the entire questionnaire was left unanswered (ie, the participant skipped the questionnaire or the participant did not log in or respond to any of the questionnaires at follow-up) or (2) items were skipped within a questionnaire that were required in order to compute a value for the total score (eg, missing items within the HADS questionnaire, which made it impossible to compute a total score from the observed data).

## Results

### Patient Characteristics

Baseline characteristics are presented in Table 1. Overall, patients were a mean age of 59.6 years (SD 8.49), and 33.5% were women (80/239). At baseline, 24.3% (58/239) reported to be receiving other forms of counseling and 18.0% (39/239) of the study sample reported taking psychotropic medications. At baseline, there was no difference between the groups in the mean HADS total score (*P*=.57).

**Figure 2:**
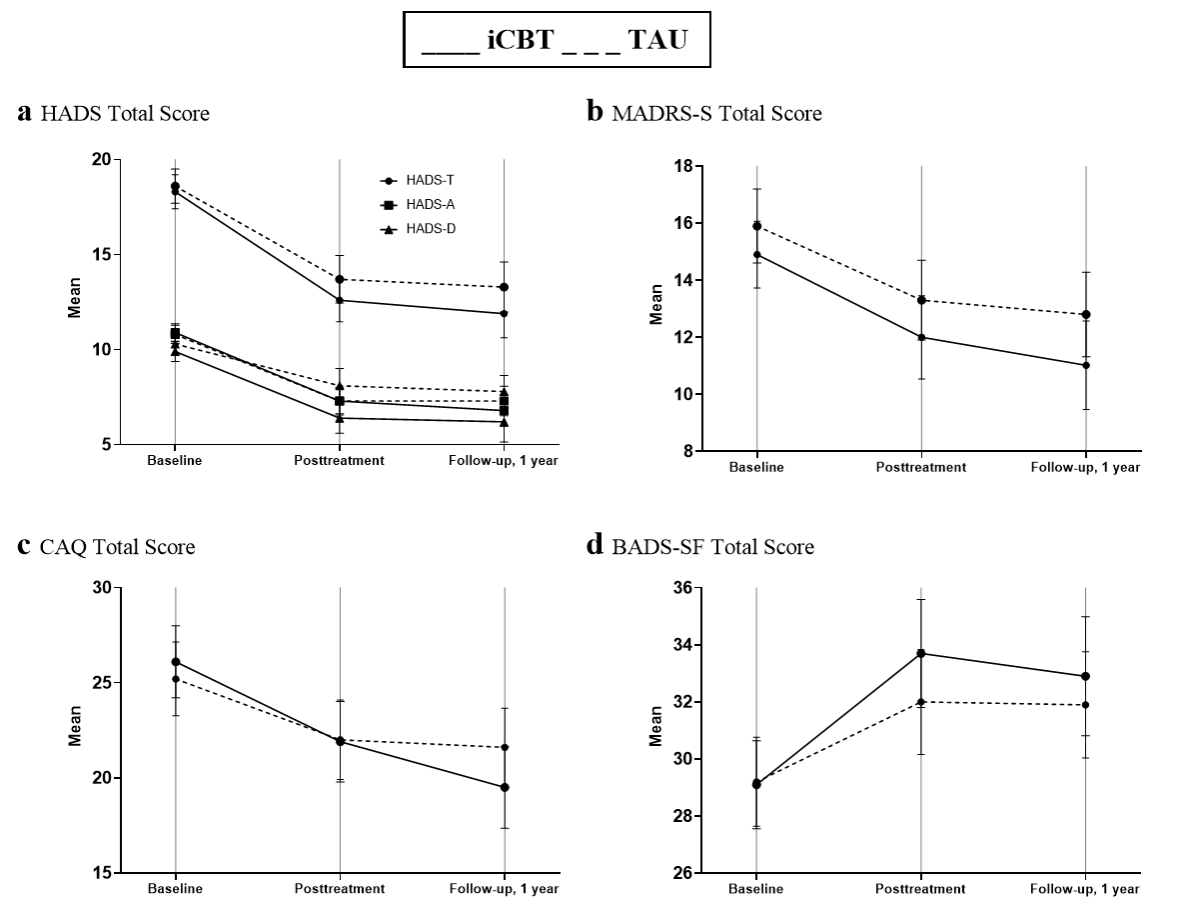
(a) Hospital Anxiety and Depression Scale total score, (b) Montgomery-Åsberg Depression Scale–Self-rated total score, (c) Cardiac Anxiety Questionnaire total score, and (d) Behavioral Activation for Depression Scale–Short Form total score mean (observed data) values at baseline, posttreatment, and 1-year follow-up. Error bars indicate 95% CI. iCBT: internet-based cognitive behavioral therapy; TAU: treatment as usual.

All primary and secondary measures (Figure 2, Multimedia Appendix 2) at posttreatment and follow-up displayed changes from baseline, indicating a reduction of self-reported anxiety or depression symptoms.

**Table 1:** Patient characteristics with observed data (ie, nonimputed).

Sociodemographic characteristics			Internet-based cognitive behavioral therapy (n=117)		Treatment as usual (n=122)
Age (years), mean (SD)			58.4 (9.0)		60.8 (7.8)
**Gender, n (%)**					
	Women	44 (37.6)		36 (29.5)	
	Men	73 (62.4)		86 (70.5)	
**Occupation, n (%)**					
	Employed	78 (66.7)		66 (54.1)	
	Unemployed	4 (3.4)		2 (1.6)	
	Retired	33 (28.2)		37 (30.3)	
	Sick leave	2 (1.7)		1 (0.8)	
	Other	0 (0.0)		2 (1.6)	
**Highest attained education, n (%)**					
	Primary school	22 (18.8)		26 (21.3)	
	Secondary school	45 (38.5)		46 (37.7)	
	University	50 (42.7)		50 (41.0)	
**In a relationship, n (%)**					
	Yes	99 (84.6)		101 (82.8)	
	No	18 (15.4)		21 (17.2)	
**Smoker, n (%)**			6 (5.1)		8 (6.6)
	Yes				
	No				
Body mass index (kg/m^2^), mean (SD)			27.8 (5.0)		27.4 (4.0)
**Taking psychotropic medicine, n (%)**					
	Yes	19 (16.2)		20 (16.4)	
	No	98 (83.8)		102 (83.6)	
**Other current counseling, n (%)**					
	Yes	30 (25.7)		28 (22.9)	
	No	87 (74.3)		94 (77.1)	
**Medical history, n (%)**					
	Myocardial infarction	19 (16.2)		13 (10.7)	
	Diabetes	21 (17.9)		19 (15.6)	
	Hypertension	42 (35.9)		51 (41.8)	
	Hyperlipidemia	26 (22.2)		27 (22.1)	
	Stroke	0 (0.0)		4 (3.3)	
	Heart failure	4 (3.4)		2 (1.6)	

### Main Analysis

HADS total scores from baseline to the 12-month follow-up visit after myocardial infarction showed a consistent reduction across the total study sample (mean difference 5.7; *t_200_*=13.57, *P*<.001). Figure 2 shows changes in mean scores over the 3 time points (baseline, posttreatment, and 12-month follow-up) of the observed (nonimputed) data.

The main model showed no difference of internet-based cognitive behavioral therapy on HADS total scores at follow-up (*β*=–1.14, 95% CI –2.73 to 0.45, *P*=.16). There was no effect of internet-based cognitive behavioral therapy on HADS depression (*β*=–1.08, 95% CI –2.31 to 0.16, *P*=.09) or anxiety (*β*=–0.64, 95% CI –1.65 to 0.37, *P*=.21) subscale scores.

### Secondary Outcomes

The CAQ was the only additional outcome to show a significant reduction at the 12-month follow-up (*β*=–2.58, 95% CI –4.75 to –0.42, *P*=.02) for internet-based cognitive behavioral therapy; however, this was not significant after adjusting for multiple comparisons (Bonferroni-corrected level of significance *P*<.008). Further details on outcomes are available in Multimedia Appendix 2.

Sensitivity analysis of observed data (*β*=–1.3, 95% CI –2.96 to 0.27, *P*=.10) and per protocol analysis (*β*=–1.3, 95% CI –2.90 to 0.41, *P*=.14) showed no effect of internet-based cognitive behavioral therapy on HADS total score.

### Cardiovascular Clinical Outcomes

Compared to those in the treatment-as-usual group, participants receiving internet-based cognitive behavioral therapy had a numerically but not significantly higher risk of experiencing a cardiovascular event (HR 1.8, 95% CI 0.96 to 3.4, *P*=.07). Posthoc analysis showed this estimate was attenuated when previous myocardial infarction and diabetes, as indicators of baseline health status, were entered in the model (HR 1.5, 95% CI 0.8 to 2.8, *P*=.25). Cardiovascular events included in the analysis are shown in Table 2.

**Table 2:** Number of patients experiencing cardiovascular events before censoring (first event), including cardiovascular-related death during follow-up.

ICD^a^ classification			Patients, n (first event, n)				
			All		Internet-based cognitive behavioral therapy		Treatment as usual
**Total**			61		36 (25)		25 (17)
	Cardiovascular-related death	2		1 (0)		1 (0)	
	Acute coronary syndrome	23		14 (13)		9 (8)	
	Stroke	3		2 (2)		1 (1)	
	Revascularization	24		12 (4)		12 (7)	
	Heart failure	9		7 (6)		2 (1)	

^a^ICD: International Statistical Classification of Diseases.

## Discussion

### Principal Findings

Primary analysis did not detect a significant difference in HADS total score between the treatment and control groups at follow-up 12 months after the myocardial infarction. These findings were in line with those of the original paper (presenting posttreatment results, ie, 14 weeks after baseline [18]). Of the secondary measures, the CAQ was the only outcome to show an effect of treatment in mean scores between the internet-based cognitive behavioral therapy and treatment-as-usual group at the 12-month follow-up, and occurrences of cardiovascular-related events did not differ significantly between the groups, although statistical power was limited.

Possible explanations to why there was no difference observed between the groups are low activity-levels among patients in the internet-based cognitive behavioral therapy arm and the low number of completed treatment modules. Just over half of those in the internet-based cognitive behavioral therapy group completed the first introductory module, and only 15% continued to work through any of the remaining 10 modules [18]. Interviews with patients from this study in a follow-up study [32] identified several factors that potentially contributed to low treatment adherence: lack of time, technical aspects (eg, insufficient computer literacy), and unpleasant emotions evoked by the intervention.

The intervention design may have contributed to low adherence. Creating an internet-based cognitive behavioral therapy intervention is technically challenging and complex and must be made with the characteristics and demographics, such as age, of the population in mind. Despite excluding individuals not able or willing to use a computer, the internet, email, or a mobile device, participants still reported low computer literacy as a contributing factor to why they did not engage more with the intervention [32]. A study [16] that reported high adherence to internet-based cognitive behavioral therapy for patients with cardiovascular disease (60% completed all modules) used nurses with experience of cardiovascular disease care to guide the intervention and suggested that this was beneficial as it allowed the participants to ask cardiovascular disease-related questions and health-related concerns. Providing a similar option for patients in our study could have likewise improved adherence. Moreover, age is one of the most well-known risk factors for myocardial infarction [33] so it is not unexpected that patients are older on average than other patient populations treated with internet-based cognitive behavioral therapy; it is important to understand how demographic characteristics of myocardial infarction patients might affect treatment engagement, particularly as it has been found that one-third of adults aged over 65 years who were average or modest internet users report feeling anxious around technology [34]. Therefore, studies with low treatment attrition and high participant satisfaction that have tailored certain aspects towards populations that may be less familiar with digital technology, should be used as design examples. For example, a recent pilot study [35] of treatment for depression in healthy older adults provided tablet-device training to the participants beforehand, required no internet connection, and used a stylus to operate the touch screen. Applying these elements to the intervention may improve results.

A well-discussed issue in intervention literature that applies to this study concerns how participants are recruited into a study that offers treatment. Patients were approached by hospital staff in participating hospitals and asked if they would be interested in participating rather than responding to an open-advertisement or actively seeking care themselves. Similar studies have also suggested that clinical recruitment might have contributed to low internet-based cognitive behavioral therapy adherence [36]. It is likely that self-referring patients would be more motivated to complete psychological treatments. A meta-analysis [37] found a more favorable effect of internet-based cognitive behavioral therapy (vs waitlist control) on anxiety outcomes for studies that deployed open community recruitment strategies as opposed to clinical service recruitment. One could speculate that this favorable difference was due to greater treatment adherence of self-referring patients and stricter inclusion and exclusion criteria.

More recent trials have had better success in maintaining participant retention, particularly when patients could self-identify as needing help with symptoms related to their coronary event and when there were fewer treatment modules to work through [19].

Of note, secondary analyses revealed significant long-term improvement on the CAQ scale in the internet-based cognitive behavioral therapy group compared to that of the treatment-as-usual group (*P*=.02). Interestingly, this parameter did not differ between groups at 14 weeks postbaseline [18]. One possible explanation might be that treatment was tailored relatively well to cardiac-specific scenarios and provided examples that were well-suited to someone with a recent myocardial infarction. Machine learning modeling with the U-CARE Heart trial data has since shown that high CAQ total score predicts participants’ adherence to the treatment [38] and that the CAQ fear subscale was the strongest predictor of participant adherence. The group differences observed on these variables might explain the intrinsic motivation of some participants to adhere more than others, and thus, may explain the findings in the CAQ outcome. Since so few participants adhered to the treatment, however, we cannot conclude that this was the reason for better CAQ scores. Previous research [4,39] has demonstrated that a treatment effect might appear at a later follow-up period despite that no effect being observed immediately posttreatment. This may explain why these effects were not present 14 weeks postbaseline. Multiple hypothesis testing can also become a problem as some results may appear by chance. After adjusting multiple comparisons, the CAQ *P* value (*P*=.02) was not significant.

The risk of cardiovascular events did not differ across groups at long-term follow-up. Unexpectedly, the hazard ratio indicated a slightly negative effect of the treatment. This effect was not significant (*P=*.07); this was a small study, with few events and lacking power for this exploratory analysis. Despite this, such an analysis is arguably justified as the knowledge gained thereof, and its potential benefits could contribute to the literature of internet-based cognitive behavioral therapy effects on cardiovascular events. Since we included a rather broad range of cardiovascular events, it is possible that those in the internet-based cognitive behavioral therapy group experienced more but milder incidences such as heart failure; the number of events were too few to adequately explore this, but upon counting the different event types in the 2 groups, no particular pattern emerged. It could also be that the treatment group was less healthy at baseline as randomization does not necessarily make groups similar. When adding indications of baseline health differences (previous myocardial infarction and diabetes), the effect was attenuated. A similar recent study [40] also found no difference in clinical outcomes between a control group and a cognitive behavioral therapy/well-being therapy group. In our study, one participant in each group died, but since neither death was the first event experienced by each respective participant, these deaths were excluded from analysis. However, as noted above, since adherence was low, it is difficult to conclude whether any effects were due to the treatment. One could speculate that it is distressing for some people to participate in a treatment study where activity is expected as it can engage feelings of guilt due to low adherence. Increased distress was the main interpretation of the negative effect on mortality in women in the nurse-led intervention in the M-HART trial [41].

### Limitations

The inclusion of participants with a rather low level of self-reported anxiety or depression (HADS anxiety or HADS depression subscale scores >7) meant that patients with only mild symptoms were included, which could be considered a disadvantage—this may have contributed to the lack of difference in the primary outcome both immediately posttreatment and at the 1-year follow-up. Spontaneous improvement over time in anxiety and depression has been observed in patients with acute myocardial infarction [42,43]. At 2 months post–myocardial infarction, it may have been too early to know which patients would still report problematic symptom levels versus those whom would recover over time, particularly as some high anxiety–reporting myocardial infarction patients have been found to steadily improve between 3 and 6 months post–myocardial infarction [44]. As previously reported [21], problems with recruitment were the initial reason for lowering the HADS anxiety and HADS depression inclusion scores from >10 to >7, and more stringent exclusion criteria (ie, a higher HADS anxiety or HADS depression threshold value for inclusion) may have allowed stronger treatment effects to be observed.

As previously reported [18], the HADS was developed largely as a screening measure for anxiety and depression and might not have been sensitive enough to detect minor changes across the measurement time points, although the inclusion of multiple secondary measures may have somewhat made up for this. Using a diagnostic interview technique is arguably one of the most robust methods to screen for anxiety and depression; however, this would have required a lot more time and therapist involvement, been more expensive, and been further from the digitalization goals around which this treatment was structured.

One challenge in this study, as is often the case in long-term follow-up studies, was the amount of missing data due to nonresponses. There was also more attrition in the internet-based cognitive behavioral therapy group than in the treatment-as-usual group at the 1-year follow-up. While this would need to be explored further to fully understand the reasons for the differences between the groups, one could speculate that it was partly because of the perceived burden by those in the internet-based cognitive behavioral therapy group of completing so many additional web-based forms and exercises, which was not experienced by those in the treatment-as-usual group. Alternatively, patients assigned to the internet-based cognitive behavioral therapy intervention could have been randomly more ill from the start, a factor which could contribute to more attrition and explain higher risk for cardiovascular events in this group.

### Conclusion

Therapist-guided internet-based cognitive behavioral therapy developed for patients with a recent myocardial infarction was not superior to treatment as usual at the 1-year follow-up in the U-CARE Heart trial; however, the CAQ scale did detect a possible trend (*P*<.02, which was not significant when corrected for multiple comparisons) toward improvement in the treatment group. This may indicate a differential effect of the internet-based cognitive behavioral therapy on cardiac anxiety that requires further research. The internet-based cognitive behavioral therapy was not associated with an increased time-to-event risk for cardiovascular events. The low adherence rates should be considered when interpreting these results.

## Appendix

Multimedia Appendix 1 Screenshot of the U-CARE Portal showing the U-CARE Heart intervention.

Multimedia Appendix 2 Outcome mean change and treatment effects at baseline, posttreatment, and 1-year follow-up.

Multimedia Appendix 3 CONSORT-eHEALTH checklist (V 1.6.1).

## Abbreviations

BADS-SF: Behavioral Activation for Depression Scale–Short Form
CAQ: Cardiac Anxiety Questionnaire
HADS: Hospital Anxiety and Depression Scale
HR: hazard ratio
ICD: International Statistical Classification of Diseases
MADRS-S: Montgomery-Åsberg Depression Scale–Self-rated
SMS: short message service
