# Supplementary figures and images for: Internet-Based Cognitive Behavioral Therapy for Patients Reporting Symptoms of Anxiety and Depression After Myocardial Infarction: U-CARE Heart Randomized Controlled Trial Twelve-Month Follow-up
Source: J Med Internet Res. 2021 May 24;23(5):e25465. doi: 10.2196/25465 (PMC8185614; doi:10.2196/25465)

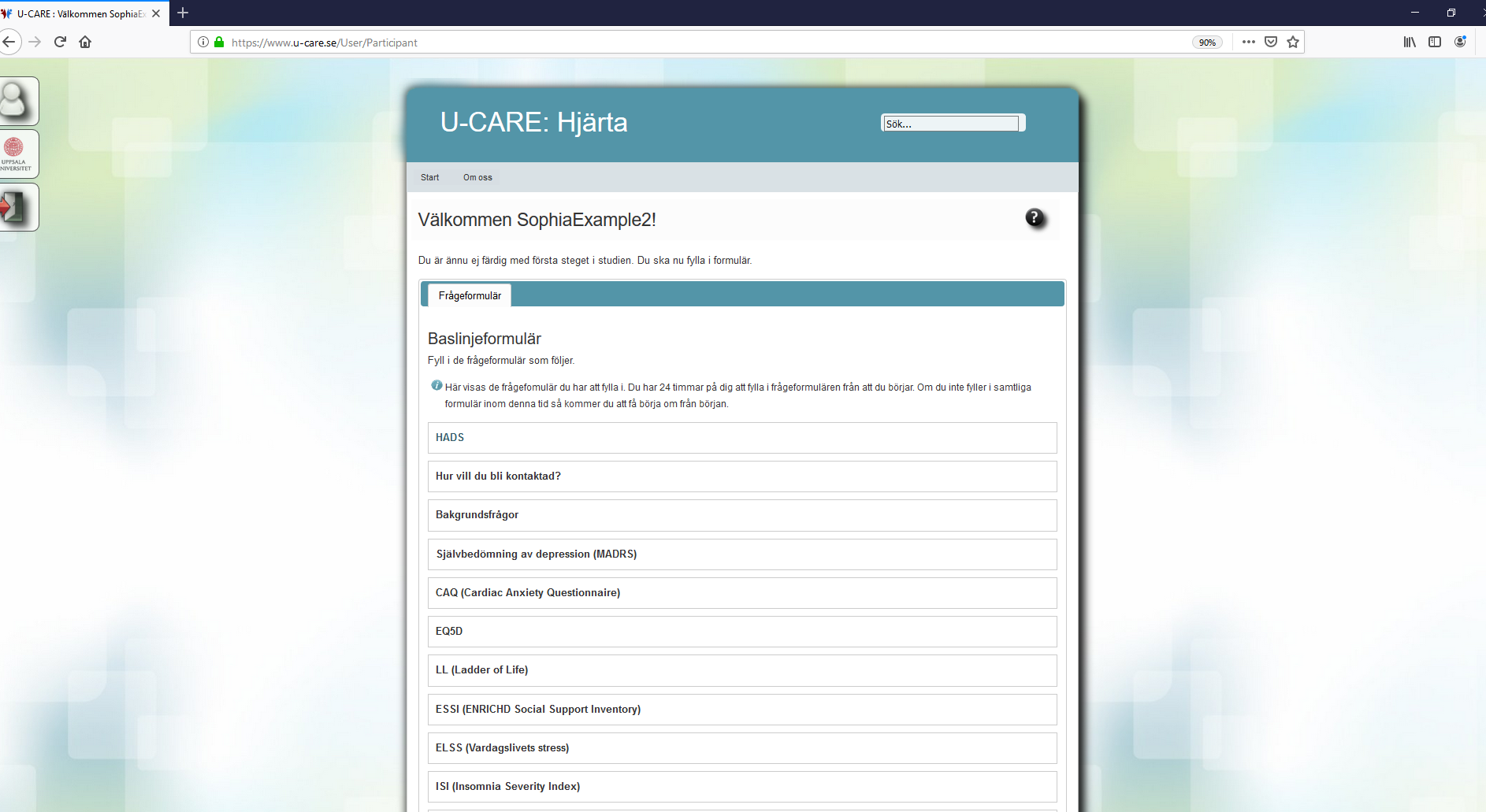

Supplement: Multimedia Appendix 1 [file jmir_v23i5e25465_app1.png]
