# Supplementary material for: Internet-Based Cognitive Behavioral Therapy for Patients Reporting Symptoms of Anxiety and Depression After Myocardial Infarction: U-CARE Heart Randomized Controlled Trial Twelve-Month Follow-up
Source: J Med Internet Res. 2021 May 24;23(5):e25465. doi: 10.2196/25465 (PMC8185614; doi:10.2196/25465)
Supplement: Multimedia Appendix 2 [file jmir_v23i5e25465_app2.docx]

**Multimedia Appendix 2.** Outcome mean change and treatment effects at baseline, posttreatment, and 1-year follow-up.

**Table 1.** Outcomes at baseline, post-treatment and 1-year follow-up, mean change and treatment effects. Mean (SD) and change are calculated from observed data. Estimate of effect (beta) are pooled adjusted coefficients for treatment versus control on imputed data. Outcome measures at post-treatment and one-year follow-up are adjusted for age, sex and baseline score on each outcome, respectively. Treatment refers to the group receiving internet-based cognitive behavioral therapy (iCBT); Control refers to standard care (SC).

| Outcome | Baseline, mean (SD) | Post-treatment, mean (SD) | One-year follow-up, mean (SD) | Change (baseline to follow-up) | Effect, Beta (95% CI) | P Value |
| --- | --- | --- | --- | --- | --- | --- |
| *HADS-T*  Treatment  Control | 18.3 (4.9)  18.6 (5.0) | 12.6 (5.6)  13.7 (6.8) | 11.9 (6)  13.3 (7) | -6.41  -5.31 | -1.14 (-2.73 to 0.45) | 0.16 |
| *HADS-A*  Treatment  Control | 10.9 (2.4)  10.8 (2.5) | 7.3 (3.1)  7.3 (3.7) | 6.8 (3.7)  7.3 (3.9) | -4.1  -3.5 | -0.64 (-1.65 to 0.37) | 0.21 |
| *HADS-D*  Treatment  Control | 9.9 (2.2)  10.3 (2.5) | 6.4 (3.0)  8.1 (3.8) | 6.2 (3.8)  7,8 (3.5) | -3.7  -2.5 | -1.08 (-2.31 to 0.16) | 0.09 |
| *MADRS-S*  Treatment  Control | 14.9 (6.4)  15.9 (7.2) | 12.0 (7.2)  13.3 (7.6) | 11. (7.2)  12.8 (7.9) | -3.88  -3.14 | -1.03 (-2.83 to 0.78) | 0.26 |
| *CAQ*  Treatment  Control | 26.1 (10.3)  25.2 (10.8) | 21.9 (10.4)  22.0 (11.3) | 19.5 (10.1)  21.6 (11) | -6.6  -3.6 | -2.58 (-4.75 to -0.42) | 0.02 |
| *BADS-SF*  Treatment  Control | 29.1 (8.4)  29.2 (8.7) | 33.7 (9.2)  32 (9.9) | 32.9 (9.5)  31.9 (9.8) | 3.8  2,7 | 0.64 (-1.67 to 2.95) | 0.59 |
